# *GExplore*: a web server for integrated queries of protein domains, gene expression and mutant phenotypes

**DOI:** 10.1186/1471-2164-10-529

**Published:** 2009-11-16

**Authors:** Harald Hutter, Man-Ping Ng, Nansheng Chen

**Affiliations:** 1 Department of Biological Sciences, Simon Fraser University, Burnaby, Canada; 2 Department of Molecular Biology and Biochemistry, Simon Fraser University, Burnaby, Canada

## Abstract

**Background:**

The majority of the genes even in well-studied multi-cellular model organisms have not been functionally characterized yet. Mining the numerous genome wide data sets related to protein function to retrieve potential candidate genes for a particular biological process remains a challenge.

**Description:**

GExplore has been developed to provide a user-friendly database interface for data mining at the gene expression/protein function level to help in hypothesis development and experiment design. It supports combinatorial searches for proteins with certain domains, tissue- or developmental stage-specific expression patterns, and mutant phenotypes. GExplore operates on a stand-alone database and has fast response times, which is essential for exploratory searches. The interface is not only user-friendly, but also modular so that it accommodates additional data sets in the future.

**Conclusion:**

GExplore is an online database for quick mining of data related to gene and protein function, providing a multi-gene display of data sets related to the domain composition of proteins as well as expression and phenotype data. GExplore is publicly available at: http://genome.sfu.ca/gexplore/

## Background

Genome sequencing projects have made available whole genome sequences of hundreds of different organisms. These valuable resources have reshaped the landscape of biology and genetics in particular. Using these genome sequences, researchers have predicted thousands to tens of thousands of genes in a typical eukaryote genome. How these genes function in an organism, however, is not immediately clear from the sequence alone. Developing better testable hypotheses requires the functional characterization of the predicted genes. This is a well recognized bottleneck for geneticists working even with the most established genetic model organisms such as the nematode *Caenorhabditis elegans*. A particular challenge is the large number of genes in any given genome in the context of the inability to quickly characterize a large number of genes in detail. Consequently the careful selection of genes for functional characterization is of particular importance in reverse genetic approaches.

*C. elegans *is one of the favorite organisms for large-scale reverse genetic screens. This is mainly due to the ability to do RNAi experiments by feeding [1] and the availability of an almost genome-wide RNAi library for such experiments [2]. Consequently genome-wide RNAi screens have been done for a number of phenotypes including survival, growth, cell division, longevity, fat storage and others [3-13]. Even though RNAi experiments are straightforward in *C. elegans *genome-wide screens are still a challenge due to the large number of genes and are effectively limited to phenotypes that can be scored quickly. Genome-wide screens completely ignore information about gene function that is already available. Selecting candidate genes using additional information available can reduce the number of genes significantly and allows screens for more sophisticated phenotypes, which tend to be more labour intensive and difficult to scale up. One example is screening for axon navigation defects, which has been done with RNAi recently, but not on a genome-wide scale [14]. Our database is designed to assist with experimental design of large-scale reverse genetic experiments in *C. elegans *in particular, since the dataset is currently limited to *C. elegans *genes.

Several lines of evidence can be used to infer the function of an uncharacterized protein. Most important are sequence similarities to known proteins, either overall similarity or at least the presence of functionally characterized protein domains. For completely uncharacterized proteins this is typically the only information available. A number of protein domain databases exist. Well established ones include ProDom [15], Pfam [16], SMART [17] and InterPro [18], which integrate a large number of data sets from various sources. All these databases have their major emphasis on the protein domains and their search and display interfaces tend to be centered on them. Consequently it is straightforward to get lists of all proteins containing a particular domain, but more difficult or impossible to do more sophisticated searches.

Additional data sets helping to elucidate gene function are expression data, either from DNA microarray experiments, SAGE experiments or even from large-scale reporter gene expression studies [19,20]. In *C. elegans *SAGE data obtained from cells and tissues purified by FACS sorting have been used to establish transcriptional profiles of the intestine [21,22], groups of neurons [23] or even individual neurons [24]. In addition stage-specific SAGE libraries have been generated [25,26]. Databases and web servers exist to probe and examine the corresponding data sets. The Stanford Microarray Database [27] is probably the most prominent site allowing users to analyse microarray data. Among other things it has been used to correlate expression patterns across a large number of microarray experiments from different species to identify genes belonging to the same pathway [28]. Gene Recommender is a novel tool, which allows researchers to exploit the microarray data set to identify genes that are regulated in a similar fashion compared to a set of candidate genes given as input [29]. The multiSAGE web site [30] allows access to the *C. elegans *SAGE data sets mentioned above. Most of these databases hold only one type of data (e.g. microarray data). Essentially only the organism-specific databases and web sites allow some access to integrated data sets. Every genome-scale experiment like a microarray experiment leaves the experimenter with a list of genes fulfilling particular experimental criteria. Usually this list of genes tends to be quite large (several hundred or even thousands of genes) and has to be narrowed down further or at least grouped for further analysis. The Gene Ontology (GO) project [31] has emerged as the quasi-standard to functionally group large sets of genes. In the absence of any other information proteins are tagged with GO terms based on protein domains with recognizable functions such as kinase domains. The GO vocabulary is rather extensive - special viewers exist to browse the vocabulary alone, which makes it difficult to use the vocabulary directly in simple interactive searches. Furthermore since many protein domains carry information about biochemical function but not biological function, the current situation with respect to meaningful functional grouping of proteins is somewhat unsatisfactory. Consequently any further analysis of large sets of genes from genome-scale experiments requires human input and intervention and therefore benefits from a simple, easy-to-use user interface.

The major integrated database for *C. elegans *genes is Wormbase [32]. Its history lies in the genome sequencing project and it has sophisticated user interfaces to access and display features at the DNA level. Data above the DNA level are organized around genes, and the major user interface at this level displays all the information and data sets related to a particular gene. Large-scale data mining and searches across different data sets is possible using a special search interface (WormMart), but the response time is slow and only selected data are accessible in this way. For many data sets at the protein level, like presence and location of protein domains, Wormbase will display the raw data from competing prediction programs, leaving the interpretation and integration to the user. This is in contrast to data at the DNA level, where the output of various gene prediction programs is integrated and only one gene model is presented. In short, even though all kinds of data related to genes and proteins are contained in Wormbase, not all data sets are equally accessible and not all are displayed in the most useful way. Missing in particular is a multi-gene interface to display data at the protein level.

A major goal of GExplore is to provide a simple and fast search and display interface that allows a multi-gene display of large data sets. Searches are generally executed within seconds. The result can be surveyed quickly and the search parameters adapted. In fact, the speed and simplicity of the output allows the researcher to quickly probe any of the underling data sets for usefulness. Researchers with their own data, e.g. a list of genes from their own genome-scale experiments, can simply paste this list of genes (up to several thousand) into the gene search field and start searching. The underlying database currently is limited to selected datasets relevant for predicting gene/protein function. It includes a search and display interface for protein domains, combined with data sets on gene expression (microarray and SAGE) and phenotype information. In addition GO terms linked to the genes are available for combinatorial searches. Currently the database is limited to *C. elegans *genes, but the overall structure is flexible enough to allow expansion of the database to incorporate data from other organisms in the future.

## Construction and content

### The user interface

#### The Individual Search Pages

GExplore contains a small set of search pages tailored towards particular types of searches. Below is a brief summary listing the pages under their menu names. Search fields for gene names and protein domains are common to all search pages except for the Literature and Compare pages. The GExplore help pages [31] contain a detailed description of all the individual search fields and display options. All search fields operating on a defined vocabulary have auto-suggest functionality, which gives a list of possible search terms as soon as the first letter is entered in the search field.

##### Domains

Contains search fields for protein domains or domain arrangements. Domain predictions are taken from the Pfam [33] and InterPro [34] databases. Allows combinatorial searches for domains as well as sophisticated domain pattern searches.

##### Phenotype

Contains text search fields to search for genes with a certain phenotype in mutants and/or RNAi experiments.

##### Expression

Contains search fields for three types of expression data: 1) full text descriptions of expression patterns; 2) selected DNA microarray experiments; 3) selected SAGE data sets

##### Combined

Combines the above search options and in addition allows to search for map position, Gene Ontology terms and homology assignment of genes.

##### Literature

A simple search interface to quickly find publications for a given list of genes. It provides links to Pubmed [35] as a way to access the publications. It covers also meeting abstracts and provides links to the full text within Wormbase [36] for those.

##### Compare

A simple interface to quickly compare two sets of genes, identifying common and unique genes in the sets.

#### The result page

Every search produces a list of genes fulfilling all search conditions. The result page displays those in a simple table format and allows manipulation of the output in various ways with a number of display options. Most display options relate to the type of data to be shown (expression data, phenotype, map position, etc). Other display options allow to sort and limit the output to a certain number of genes and to remove individual genes manually in order to fine-tune the output.

### The underlying database

Searches are executed by querying a local MySQL database [see Additional file 1 for the database schema]. Data contained in this database were ultimately derived from other public databases and the primary literature. Currently Wormbase [36] and multiSAGE data [30] as well as data directly extracted from primary literature are used as primary data source. Raw data related to gene/protein function were downloaded, processed and organized in a local database. Processing is aimed towards a meaningful simplification and integration of data. It provides an essential distinction over existing databases and currently includes the following:

#### Genes and Proteins

Splice variants are intentionally ignored, only the longest splice variant is used for display. Consequently each gene is represented in the output exactly **once **and the number of retrieved proteins is equivalent to the number of different genes fulfilling the search criteria. Links to Wormbase are provided for researchers interested in any particular gene in detail.

#### Protein domains

Protein domains are structurally and functionally defined regions of a protein typically inferred from recognizable sequence similarities. Several databases provide this kind of analysis, Interpro [34] and Pfam [33] annotations are used here. About 270 domain currently have individual abbreviations and symbols for display. Redundant Interpro and Pfam domains were combined and are represented using the same abbreviation. Domain abbreviation as well as Interpro and Pfam identifiers can be used for searching. Several rules have been implemented to eliminate redundant domains and to resolve conflicting domain predictions such that there is **only one **domain displayed for any given part of a protein and **only one **display per protein. Redundant Interpro domains essentially predicting the same domain are collapsed into a single abbreviation to simplify the display. Note that Interpro domain identifiers (e.g. IPR0013149) can always be used explicitly as well for searches, so that collapsing them for display purposes does not prevent more specific searches. Certain 'Domains' essentially define protein families (like Cytochrome_P450, Globin, Innexin). They are shown as large rectangles in the display. These 'meta-domains' tend to cover entire proteins and potentially obscure real proteins domains (like transmembrane domains (TM) in the case of Innexins). Underlying domains are still available for searching, i.e. searching for 'TM' will retrieve innexins, even though the TM is not shown in the domain display of the protein.

The following priority rules establish which domain is displayed when annotations overlap:

Rule 1: domains completely embedded in other domains are not shown.

Rule 2: N-terminal signal sequences are always displayed

Rule 3: Transmembrane domains have priority, i.e. are displayed even when embedded in other domains

Rule 4: meta-domains have top priority, i.e. are always shown

#### Rationale and explanation

Frequently domains have multiple Interpro domains associated with it causing multiple overlapping domain predictions (IG domains and EGF modules are particularly good examples for this, see **UNC-52 **as example). Rule 1 collapses these. Since Interpro unfortunately has many large 'domains' that are not really protein domains, rule 1 tends to collapse too much, which leads to a series of rules as to which Interpro domains should be ignored (see below). Signal sequenes and transmembrane domains are important indicators for the localization of a protein and have therefore precedence.

The following rules suppress the display of certain Interpro domains:

Rule 5: Any domain overlapping a signal sequence is suppressed. Domains that partially overlap and extend for more than 30 amino acids outside the signal sequence are displayed after the signal sequence. Explanation: Signal sequences are characterized by a hydrophobic core, which sometimes is separately predicted as transmembrane domain. Certain domain predictions extend into and therefore overlap signal sequences (e.g. the CW domain).

Rule 6: any domain that covers more than 90% of a protein or is longer than 300 amino acids is ignored (with the exception of a few domains that genuinely seem to be larger than 300 amino acids). The rule does not apply to meta-domains. Explanation: protein domains in the sense of 'structurally and functionally defined regions of a protein' tend to be between 30 and 150 amino acids long. Anything shorter is too short to be an independent self-folding unit and anything larger typically can be subdivided further. Very few protein domains in this sense fall outside this range. Interpro contains both shorter and larger 'domains'. Catalytic cores of enzymes, phosphorylation sites or other small protein motifs are generally not displayed here. Certain large Interpro domains, which are essentially diagnostic for particular protein families are considered meta-domains and are displayed.

Rule 7: Interpro domains smaller than half or larger than twice the average size of the domain are ignored. Explanation: This effectively suppresses partial domain predictions and tries to deal with the problem that some Interpro domains (like some of IG domains) effectively fuse several domains of the same type (which are frequently correctly predicted by redundant other Interpro domains or by Pfam).

Taken together these rules effectively suppress the display of certain domains with the ultimate goal of creating a simple yet meaningful output similar to domain displays found in publications of individual characterized proteins. These rules are only applied at the display step and do not affect searches and retrieval of proteins.

#### SAGE data

SAGE data are quantitative expression data. Briefly, small sequence tags are generated from mRNA samples and sequenced in large numbers. Tags are mapped to the genome to identify the genes present in the original sample. Normalized tag counts can be used to compare expression levels of genes across several samples. The SAGE data used here were downloaded from the multiSAGE web site [30] and processed in the following way: 1) only tags unambiguously mapped to coding mRNA were used and 2) all tags belonging to the same gene were added up. SAGE data are presented in logical groups (e.g. embryonic tissues or life stages) and can either be displayed as normalized tag counts or as enriched/depleted with respect to a reference library.

#### Microarray data

Selected DNA microarray data sets were extracted from the literature [21,37-43]. Data sets were selected for general usage (preference for expression profiling of tissues over more specific sets) and date of publication (preference for recent data sets due to difficulties of mapping older sets to current gene predictions). Each of these data sets essentially is a list of genes fulfilling a certain condition ('expressed in neurons' or 'enriched in muscle'). From a database and search perspective this translates into a simple tag for the genes in the set. The list of tags used can be found on the microarray help page of the website. All these tags are accessible from a single search field with Boolean search logic. This allows simple comparisons of data sets across different publications and comparison with other data sets like SAGE data or completely unrelated data.

#### Other data

The remaining data on this site (concise description of genes, phenotype and expression description, Gene Ontology terms) have been extracted from Wormbase with little processing. All terms (descriptions, etc) belonging to the same gene have been integrated and are presented as single entry.

## Utility and Discussion

GExplore is a tool for large scale mining of data related to gene or protein function. It is currently limited to *C. elegans *genes. The interface is simple and response times are fast, encouraging exploratory searches and quick fact checking. This site should be useful to plan of genome-scale experiments and survey-type queries related to gene and protein function. Researchers with their own data, e.g. a list of genes from their own genome-scale experiments, can simply paste this list of genes (up to several thousand) into the gene search field and start searching.

With this interface you should be able to get prompt answers to questions like: I need ...

- a list of small secreted proteins (candidate signaling molecules)

- putative cell surface receptors expressed in neurons

- kinases expressed in muscle cells with mutants available (and their phenotypes)

- secreted or transmembrane proteins with LRR domain and their recent publications

**Figure 1:**
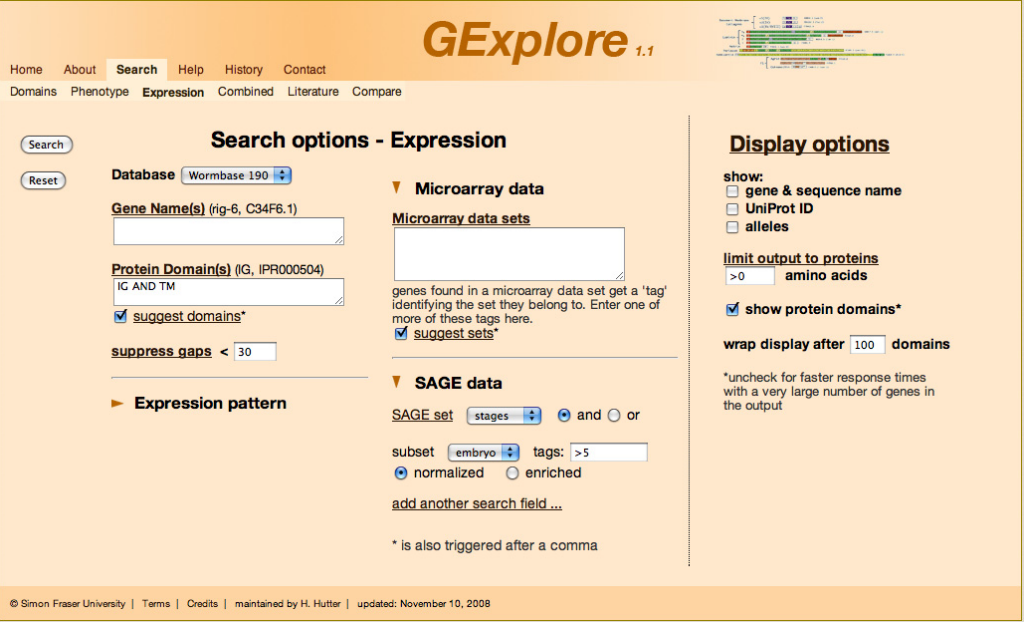
**Sample search interface**. The figure shows the search interface set up for a search of transmembrane proteins containing immunoglobulin repeats that are expressed in the embryonic SAGE library with at least 5 tags per 100.000 tags.

**Figure 2:**
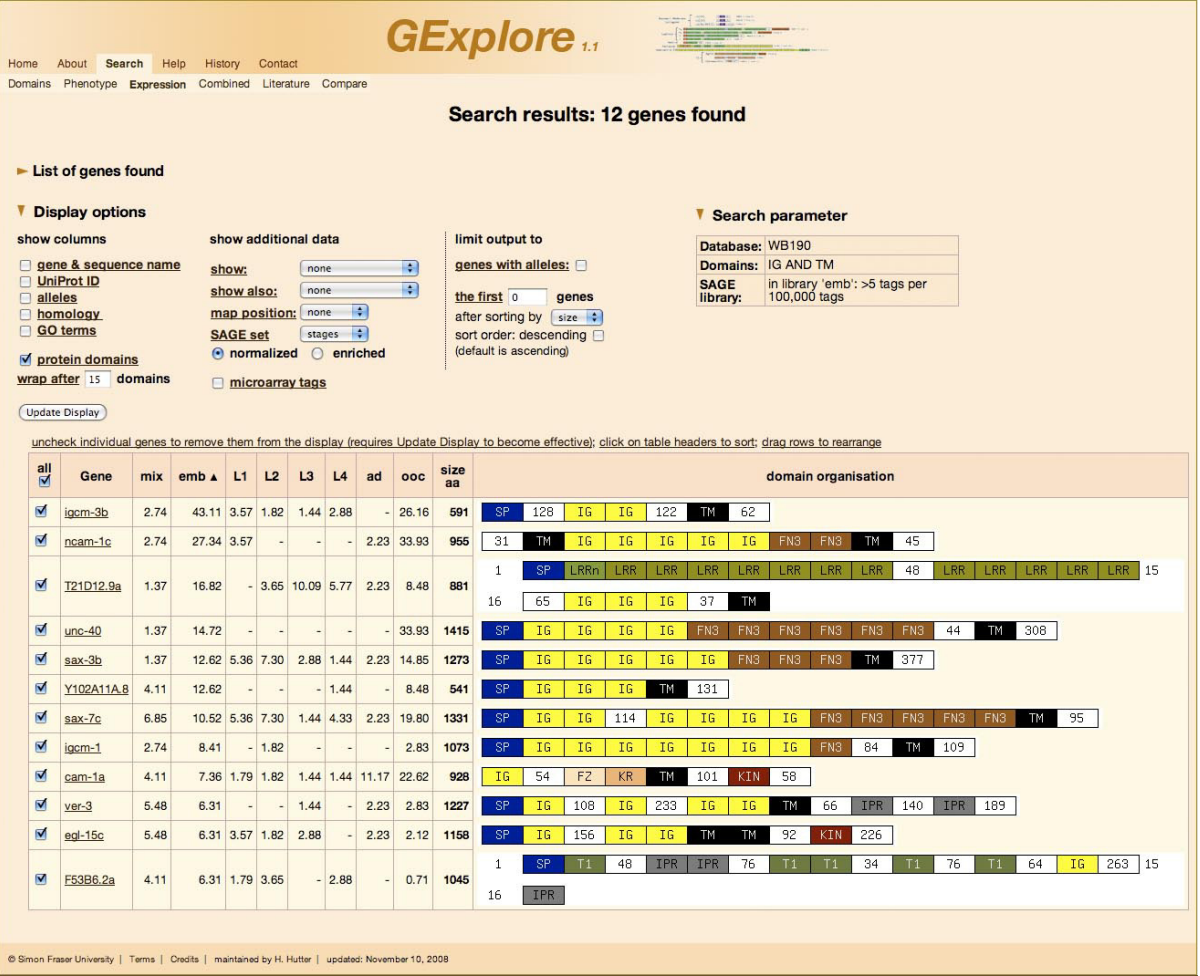
**Output page**. The figure shows the output of the search shown in Figure 1 after sorting the output for the highest number of tags in the embryo library.

A sample search is shown in Figure 1. The figure shows the search interface set up for a search for transmembrane proteins containing immunoglobulin repeats that are expressed in the embryonic SAGE library with at least 5 tags per 100.000 tags. Figure 2 shows the corresponding output, after sorting for the highest number of tags in the embryo library.

### Protein domain display

Several web interfaces provide a display of the domain organization of proteins. Among the most prominent ones are ProDom [44] and SMART [17]. Other protein domain databases like Pfam [33], Prosite [45] or Interpro [34] also have some capability to display the domain organization of individual query proteins or all proteins containing a certain domain. For a biologist/geneticist trying to get an overview of proteins of a particular organism, these web interfaces pose some challenges. First of all, all these databases use protein database identifiers (e.g. Q6W3C6_CAEEL) rather than the familiar name (FMI-1) on the input side (search fields) as well as on the output side. This complicates phrasing queries and interpreting the output. Secondly, these databases typically operate on redundant protein databases, possibly containing multiple copies of the same protein like splice variants or protein fragments. While this is desirable for the sake of completeness, it is not helpful for queries, where details about any individual gene are less important than the exact number of genes fulfilling the search condition. Finally, all these databases are specialized in the sense, that they only contain one type of information (protein domain information), which cannot be combined with other information like expression or phenotypic data for search and display.

### Organism-specific web sites

Web sites and databases dedicated to particular organisms generally do not have the disadvantages mentioned above: they use the common gene and protein names and contain combined expression and phenotypic information about genes. Model organisms like *C. elegans*, *Drosophila *or mouse have well established sites (Wormbase [36], Flybase [46], Mouse Genome Informatics [47]) which are updated and maintained by large groups of dedicated bioinformaticians. These are complex sites with multiple search interfaces. However, they have other disadvantages, most notably they tend to be single gene-centered, i.e. do not provide multi-gene output and they typically do not provide a convenient protein domain display. Since the majority of the genes and proteins are still uncharacterized and since the presence of certain protein domains is a strong predictor of function, this is a handicap for geneticists interested in large scale data-mining and comparison of previously uncharacterized genes.

### The niche for GExplore

GExplore is an attempt to fill this gap and provide a fast search and display interface for data related to gene expression and function. GExplore provides a simple multi-gene display on the output side, which allows the user to quickly scan through information on larger sets of genes. This sets it apart from more comprehensive databases like Wormbase. GExplore provides easy access to datasets like SAGE data, which are included in Wormbase, but essentially not accessible due to the lack of a suitable interface. Datasets, which are presented as raw data, like SAGE data and Interpro and Pfam annotations are processed and integrated in GExplore to provide a user-friendly display of expression and domain organization. Search fields, which operate on a defined vocabulary, like the Gene Ontology terms [31], have 'auto-suggest' functionality, where possible search terms are suggested upon typing the first letters. This enables users without knowledge of the vocabulary to access the data without having to learn the vocabulary first. In combination these features complement existing databases and make GExplore most useful for planning of large-scale experiments related to probing gene function.

## Conclusion

GExplore is a web interface for quick mining of data related to gene and protein function, providing access to data sets relating to domain composition of proteins as well as expression and phenotype data.

## Availability and requirements

The web site is hosted under http://genome.sfu.ca/gexplore/ and can be used with all major browsers. Certain features require Javascript to be enabled in the browser.

## Authors' contributions

M-P.N. implemented the domain display interface under supervision of N.C. H.H. conceived the project, implemented the remaining parts and wrote the manuscript together with N.C. All authors read and approved the final manuscript.

## Supplementary Material

Additional file 1 **GExplore database schema**. Database schema and description of data fields. Click here for file
