# Supplementary material for: GExplore: a web server for integrated queries of protein domains, gene expression and mutant phenotypes
Source: BMC Genomics. 2009 Nov 16;10:529. doi: 10.1186/1471-2164-10-529 (PMC2779824; doi:10.1186/1471-2164-10-529)
Supplement: Additional file 1 — GExplore database schema. Database schema and description of data fields. [file 1471-2164-10-529-S1.PDF]

GExplore database schema

domainPattern
gene varchar(20)
CDS varchar(20)
domains varchar(1000)
pattern varchar(5000)
meta_pattern varchar(5000)

literature
ID varchar(30)
WB_paperID varchar(30)
PubMed_ID varchar(30)
type varchar(100)
year varchar(30)
authors varchar(1000)
citation varchar(1000)
keywords varchar(1000)
gene varchar(30)
gene_public varchar(30)

genes
geneWBID varchar(30)
gene varchar(20)
CDS varchar(20)
CGC varchar (10)
wormpepID varchar (20)
uniprot varchar(20)
protsize int(10)
chromosome varchar(10)
cM float(20)
strand varchar(1)
startPos int(10)
endPos int(10)
alleles varchar(500)
transposons varchar(500)
homology varchar(50)
GO_IDs varchar(200)
GO_terms varchar(1000)
mutant_phenotype varchar(1000)
RNAi_phenotype varchar(1000)
expression varchar(10000)
stages varchar(1000)
anatomy_terms varchar(1000)
expression_tags varchar(1000)
concise_description varchar(10000)

SAGE
gene varchar(20)
mixNorm float(10)
embNorm float(10)
L1Norm float(10)
L2Norm float(10)
L3Norm float(10)
L4Norm float(10)
adNorm float(10)
oocNorm float(10)
mixEnrich float(10)
embEnrich float(10)
L1Enrich float(10)
L2Enrich float(10)
L3Enrich float(10)
L4Enrich float(10)
adEnrich float(10)
oocEnrich float(10)
embrNorm float(10)
phaNorm float(10)
gutNorm float(10)
musNorm float(10)
hypNorm float(10)
neuNorm float(10)
embrEnrich float(10)
phaEnrich float(10)
gutEnrich float(10)
musEnrich float(10)
hypEnrich float(10)
neuEnrich float(10)
embryoNorm float(10)
AFDNorm float(10)
ASERNorm float(10)
cilNorm float(10)
unc4Norm float(10)
embryoEnrich float(10)
AFDEnrich float(10)
ASEREnrich float(10)
cilEnrich float(10)
unc4Enrich float(10)

Supplementary data: Description of database fields

Table: genes (main table)

Field	Content
geneWBID	Wormbase gene ID (e.g. WBGene00016354)
gene	gene name (e.g. C33F10.5)
CDS	protein identifier (e.g. C33F10.5b)
CGC	CGC gene name (e.g. rig-6)
wormpepID	wormpep protein ID (e.g. CE25803)
uniprot	Uniprot identifier (e.g. Q9BIA2)
protsize	protein size
chromosome	chromosome
cM	genetic position
strand	strand orientation
startPos	physical position of gene start
endPos	physical position of gene end
alleles	all alleles
transposons	transposon insertion in the gene
homology	condensed homology information
GO_IDs	GO term identifiers
GO_terms	GO terms
mutant_phenotype	all mutant phenotypes
RNAi_phenotype	all RNAi phenotypes
expression	combined expression information
stages	stages of expression
anatomy_terms	anatomy terms associated with expression
expression_tags	expression tags generated from microarray data sets
concise_description	concise gene description (see wormbase data field)

Table: literature

Field	Content
ID	unique number
WB_paperID	Wormbase identifier (e.g. WBPaper00000031)
PubMed_ID	PubMed identifier (e.g. 4366476)
type	type of publication (e.g. ARTICLE)
year	year of publication
authors	list of authors
citation	citation (as in wormbase)
keywords	keywords associated with the publication
gene*	gene associated with the publication (e.g. F41C6.1)
gene_public	CGC gene name (e.g. unc-6)

*note: this field contains only one gene name. If a publication is associated with several genes, separate entries for each gene will be generated.

Table: SAGE

Field*	Content
gene	gene name (e.g. C33F10.5)
mix, emb, L1, L2, L3, L4, ad, ooc	SAGE data for the different stages
embr, pha, gut, mus, hyp, neu	SAGE data for the different tissues
embryo, AFD, ASER, cil, unc4	SAGE data for different neuronal subpopulation

*note: fields ending in 'Norm' contains normalized SAGE tags, fields ending in 'Enrich' contain the enrichment factor with respect to the reference library

Table: domainPattern

Field	Content
gene	gene name (e.g. C33F10.5)
CDS	protein identifier (e.g. C33F10.5b)
domains	all domains found in the protein
pattern	sequence of domains in the protein (excluding metapattern*)
meta_pattern	sequence of domains in the protein

*note: currently not used. Could be used to remove metadomains from the display to show the underlying domains (like transmembrane domains)
